# Gain in polycrystalline Nd-doped alumina: leveraging length scales to create a new class of high-energy, short pulse, tunable laser materials

**DOI:** 10.1038/s41377-018-0023-z

**Published:** 2018-07-04

**Authors:** Elias H. Penilla, Luis F. Devia-Cruz, Matthew A. Duarte, Corey L. Hardin, Yasuhiro Kodera, Javier E. Garay

**Affiliations:** 1 Advanced Materials Processing and Synthesis (AMPS) Laboratory, UC San Diego, La Jolla, CA 92093 USA; 2 Materials Science & Engineering and Mechanical & Aerospace Engineering, University of California, San Diego, La Jolla, CA 92093 USA

## Abstract

Traditionally accepted design paradigms dictate that only optically isotropic (cubic) crystal structures with high equilibrium solubilities of optically active ions are suitable for polycrystalline laser gain media. The restriction of symmetry is due to light scattering caused by randomly oriented anisotropic crystals, whereas the solubility problem arises from the need for sufficient active dopants in the media. These criteria limit material choices and exclude materials that have superior thermo-mechanical properties than state-of-the-art laser materials. Alumina (Al_2_O_3_) is an ideal example; it has a higher fracture strength and thermal conductivity than today’s gain materials, which could lead to revolutionary laser performance. However, alumina has uniaxial optical proprieties, and the solubility of rare earths (REs) is two-to-three orders of magnitude lower than the dopant concentrations in typical RE-based gain media. We present new strategies to overcome these obstacles and demonstrate gain in a RE-doped alumina (Nd:Al_2_O_3_) for the first time. The key insight relies on tailoring the crystallite size to other important length scales—the wavelength of light and interatomic dopant distances, which minimize optical losses and allow successful Nd doping. The result is a laser gain medium with a thermo-mechanical figure of merit of *R*_s_~19,500 Wm^−1^ a 24-fold and 19,500-fold improvements over the high-energy-laser leaders Nd:YAG (*R*_s_~800 Wm^−1^) and Nd:Glass (*R*_s_~1 Wm^−1^), respectively. Moreover, the emission bandwidth of Nd:Al_2_O_3_ is broad: ~13 THz. The successful demonstration of gain and high bandwidth in a medium with superior *R*_s_ can lead to the development of lasers with previously unobtainable high-peak powers, short pulses, tunability, and high-duty cycles.

## Laser materials: Processing tricks help increase sapphire’s appeal

Transparent ceramics doped with rare-earth atoms have mechanical properties that may help surpass outputs of conventional solid-state lasers. Because laser power is a function of a materials’ thermal conductivity and shock resistance, researchers have investigated robust sapphire crystals as potential platforms for high-energy lasers. Javier Garay and co-workers at the University of California San Diego now report a process that uses elevated heating and cooling rates to incorporate neodymium dopants into sapphire to achieve, optical gain which is necessary for laser amplification. The technique relies on fabricating ceramics composed of nanoscale crystallites, smaller than the laser light wavelength. These nanostructures reduce unwanted light scattering from sapphire’s naturally asymmetric atomic structure, and ensure even dopant distribution. The large, tunable bandwidth of the new sapphire laser could aid applications needing ultra-short, high-power pulses.

## Introduction

The past decade has seen significant advances in the development of high-energy laser (HEL) technologies, with improvements in pumping technology, cavity design, cooling methods, and gain media quality. The search for gain media with superior optical, thermal, and mechanical properties remains intense because improvements in the material properties translate directly to increases in device performance^1, 2^. Advanced laser gain materials that provide access to wavelength tunability, short pulses, and so on have paved the way for the study of light-matter interactions^3–6^, break-through medical applications^7^, and imaging/spectroscopy^8^.

Single crystals and glasses dominate the gain media market, but recent pioneering efforts have revealed advantages of polycrystalline ceramics such as improved mechanical properties and the possibility of gradient doping^9^. Ceramics also have the potential to alleviate one of the most pressing challenges in solid-state lasers—the thermal management of gain media. The power deliverable by a laser scales directly with thermal conductivity *k*, and the fracture stress *σ*_F_ places an ultimate limit of failure such that the figure of merit for a gain material is given by

1 $$R_{\rm{s}} = \frac{{k(1 - v)}}{{\alpha E}}\sigma _{\rm{F}}$$

where *E* is the elastic modulus, *α* is the coefficient of thermal expansion, and *v* is Poisson’s ratio. The low thermal conductivities of leading gain media (~1–2 Wm^−1^ K^−1^ RE:Glass^10^ and 7–14 Wm^−1^ K^−1^ RE:YAG^11^) continue to limit the power scaling of HELs.

Encouraged by pioneering work on cubic (optically isotropic) YAG ceramics that demonstrated lasing performance that rival their single-crystal counterparts^12–14^, researchers have been working on other cubic materials^15–18^ as RE-host media because they have higher *k* than YAG^18, 19^. Cubic-symmetry materials such as garnets and RE-sesquioxides are the mainstay of transparent ceramics because grain growth need not be avoided to mitigate birefringence scattering, and they readily accommodate RE dopants due to the similarity in ionic radii between dopant and cations^20^. The advances have been significant, but the improvements in thermo-mechanical characteristics have been insufficient to rival the state-of-the art gain media. To supplant RE:Glass and/or RE:YAG, a gain material with substantially better thermo-mechanical properties is needed.

For decades, researchers have worked on developing sapphire/alumina as a RE host because Al_2_O_3_ offers superior thermal conductivity (*k*~30–35 Wm^−1^ K^−1^)^21^ and a high-fracture toughness (3.5 MPam^−1/2^)^22^, the combination of which leads to a superior thermal shock resistance (*R*_s_~19,500 Wm^−1^) compared to that of Glass (*R*_s_~1 Wm^−1^)^23^ and YAG (*R*_s_~800 Wm^−1^)^1, 24^. Moreover, sapphire has a long history as a transition metal-doped gain media. Ruby (Cr:Al_2_O_3_) was the material used in the first laser^25^, and even today, titanium sapphire (Ti:Al_2_O_3_) is the most pervasive tunable laser medium^26^. The addition of RE dopants at levels sufficient for gain could allow for efficient emission at other wavelengths, resulting in a laser gain medium with a combination of thermal, mechanical, and optical properties that will lead to more powerful lasers for scientific, medical, industrial, and mobile applications.

Despite these promising attributes, producing laser grade RE:Al_2_O_3_ ceramics is usually thought of as impossible. The two main obstacles are (1) the disparity in ionic radii between the RE^3+^ and Al^3+^, which leads to an equilibrium solubility of ~10^−3^%^27^, which is lower than necessary for gain, and (2) the optical anisotropy arising from the hexagonal crystal structure of Al_2_O_3_ leads to birefringence scattering that must be mitigated to achieve high transparency.

There have been significant efforts in developing powders^28, 29^ and thin films^30–34^. Rand, Laine, and co-workers demonstrated the promising result of random lasing in strongly scattering rare-earth doped δ–Al_2_O_3_ powders using direct electron-beam pumping^28, 29^. Significant progress has also been made in Er^3+^ and Er^3+^/Yb^3+^ doped alumina thin films fabricated by RF-magnetron sputtering^30^ and pulsed laser deposition (PLD)^31, 32^ to concentrations as high as 1 at.%, which resulted in amorphous and/or mixtures of amorphous and crystalline films with measurable photoluminescence (PL). Recently, Waeselmann et al. reported lasing in ~2.6 μm single-crystal Nd:Sapphire thin films and reported dopant concentrations of ~0.3–2at.%^35–37^. These reports are encouraging for producing lasers from RE:Al_2_O_3_ media, but because of the low thermo-mechanical properties of powders and the difficulty in scaling thin films, they are not practical for HELs.

Translucent alumina ceramics have been produced for decades^38, 39^, but there are only a few reports on bulk ceramic RE:Al_2_O_3_. Importantly, gain has not been demonstrated because RE:Al_2_O_3_ ceramics have not reached the necessary optical quality^19, 40, 41^. Krebs and Happek^40^ used a laser-heated pedestal growth (LHPG) approach to produce single-crystal Yb^3+^:Al_2_O_3_ fibers, and Sanamyan^41^ et al. used a combination of powder synthesis and CAPAD to form dense Er^3+^:Al_2_O_3_. In both instances, single-site doping of RE onto the Al^3+^ lattice was possible at concentrations below the RE solubility limit, but at higher concentrations, secondary phases that hindered PL formed. It remains unclear whether these materials possess sufficient PL and low losses necessary for gain/lasing.

In our previous work^19^, we first reported PL in the visible with long lifetimes in transparent polycrystalline Tb^3+^:Al_2_O_3_. While promising for the feasibility of using RE:Al_2_O_3_ ceramics as a gain media, we did not show evidence of stimulated emission or optical gain.

In this work, we present the first bulk polycrystalline Nd:Al_2_O_3_ ceramics that exhibit stimulated emission and optical gain. Importantly, we demonstrate that gain can be achieved without single sight doping, i.e., with some Nd segregated to the grain boundaries. We report for the first time the presence of absorption bands in the transmission spectra, which confirm the presence of optically active Nd^3+^ present within the ceramic matrix. For the primary pumping band at 806 nm (^4^I_9/2_ → ^4^F_5/2_), the absorption cross-section is 1.36 × 10^−20^ cm^2^ and 1.69 × 10^−20^ cm^2^ for 0.25 at.% and 0.35 at.% Nd:Al_2_O_3_ ceramics, respectively.

In addition to the thermal management problem, Nd:Al_2_O_3_ addresses another long-standing problem in HEL technologies—producing broadband emission in RE-doped media. Conventional gain media design aims for sharp single-site peaks that result in lower lasing thresholds. The advantage of high bandwidth is wavelength tunability and/or the generation of short pulses (increased peak energy). When pumping at 806 nm the ceramics show a 50 nm (FWHM), 13 THz peak at 1064 nm, (^4^F_3/2_ → ^4^I_11/2_). The fluorescence lifetime is ~150 μs, which results in stimulated emission cross-sections as high as ~9.8 × 10^−21^ cm^2^. The 13 THz gain bandwidth arising from multi-site doping of Nd in Al_2_O_3_ is a new record for Nd^3+^ gain media and could lead to pulses as short as 8 fs. Importantly, the measured gain coefficient, *g*_*o*_, is as high as 2.42 cm^−1^ for 0.35 at.% Nd^3+^:Al_2_O_3_ at 1064 nm. The combination of thermal, mechanical, and optical properties offered by Nd^3+^:Al_2_O_3_ opens the door to producing HELs with superior performance. Moreover, the approach presented is applicable to other anisotropic material systems that are not readily considered for optical applications.

## Results

**Fig. 1:**
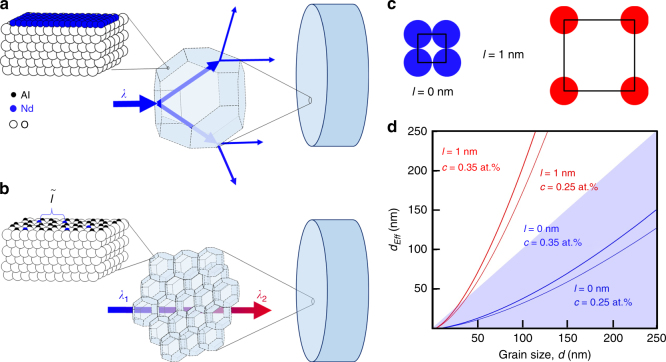
Length scale relationships important for achieving gain in anisotropic ceramics. **a** Light is scattered at grain interfaces in ceramics with large crystallites because randomly oriented grains represent discontinuities in refractive index. RE segregation (represented as a close-packed monolayer) at the grain boundary on a section of Al_2_O_3_ (the blue atoms are Nd, those in white are O, and those in black are Al). **b** Scattering efficiency decreases significantly when pump (*λ*_1_) and emitted light (*λ*_2_) wavelengths are smaller than the grain size, permitting low optical losses. Small grains also permit spreading out of RE dopants at grain boundaries, increasing average interionic distance,$\tilde l$ allowing for optical gain. **c** A close-packed arrangement of dopant *l* = 0 and one with realistic interionic distance for gain (*l* = 1 nm). **d** Calculation of grain size necessary to accommodate all the dopants for a given dopant arrangement and concentration on the grain boundary, *d*_eff_ vs. grain size using Eq. 4 for two concentrations and arrangements shown in (**c**)

Our strategy for obtaining gain in Nd:Al_2_O_3_ is a twofold design of nano/microstructure that relies on (1) crystallite sizes below the wavelength of pump and emitted light and (2) a dopant distribution in the grain volumes with minimal segregation at the grain boundaries. Figure 1 summarizes our strategy. In anisotropic ceramics with large grains, light is scattered at grain interfaces since they represent discontinuities in refractive index (Fig. 1a). However, as grain size decreases, the scattering efficiency of uniaxial grains is significantly lower^38, 39, 42^. Thus, fine-grained ceramics can be highly transparent media with losses that are low enough to achieve optical gain (Fig. 1b).

In addition to low losses, RE-dopant concentrations must be within a critical range—high enough to achieve a sufficient absorption cross-section and emission cross-section, yet low enough to prevent concentration quenching (energy relaxation through phonon rather than radiative photon processes), which occurs when ions are too closely spaced.

Traditional material processing can be employed in systems such as glasses and garnets where RE solubility is high. However, in low solubility media, agglomeration occurs at grain boundaries (as shown in Fig. 1a). In the isotropic laser ceramics that have been demonstrated, grain sizes are typically 10–20 μm^14^. In this large grain size case, there are relatively few grain boundary regions to accommodate the RE-dopant, and the average distance between RE ions decreases, resulting in luminescence quenching.

A key insight here is that the fine crystallite sizes that allow for high transparency in anisotropic polycrystalline materials can also play a crucial role in absorption/emission by providing a possibility for higher RE incorporation without luminescence quenching. By reducing grain size, grain boundary volume increases. When holding the global dopant concentration constant while decreasing grain size, RE dopants can ‘spread out’ along grain boundaries, increasing the average distance $\tilde l$ between RE- ions (Fig. 1b). In other words, for very fine-grained materials, it should be possible to reach dopant concentrations sufficient to achieve gain even without solubility in the grain interior. The effective grain size *d*_eff_ necessary to accommodate all the dopants on the grain boundaries rather than in the grain interiors depends on the arrangement of dopants on the boundary (function of $\tilde l$) and scales with *d*^3/2^ (see the Materials and methods for details).

To illustrate this scenario, we plot *d*_eff_ as a function of grain size (Eq. 4) in Fig. 1d for various concentrations (at.% Nd) and dopant arrangements (Fig. 1c). The shaded regions in Fig. 1d are conditions in which it is possible to accommodate the global concentration of dopant atoms *c* without any solubility in the grain. In the non-shaded regions, *d*_eff_ > *d*, meaning that it is not possible to accommodate all the dopant ions without solubility in the grains. In the limiting case example of a close-packed monolayer ($\tilde l$ = 0) (Fig. 1c), it is possible to accommodate *c* = 0.25 at.% and *c* = 0.35 at.% of Nd on the grain boundary of a grain with *d*~250 nm. The close-packed monolayer case would likely not lead to gain because the distance between RE ions would result in luminescence quenching. Using a realistic value of $\tilde l$=1 nm, we see that grain sizes <25 nm are necessary to accommodate 0.35 at.% of Nd. The need for such small grain sizes is alleviated in our case because alumina does have solubility in the grain interiors which is likely higher near grain boundaries and can be increased under specific processing conditions as will be discussed below. It is interesting to discuss this level of dopant incorporation relative to Nd:YAG. The high Nd equilibrium solubility in YAG is due to the more open crystal structure leading to a lower cation density compared to that for alumina. Because the cation density is higher in Al_2_O_3_, the volume concentration, *c*_vol_, of Nd is significantly higher in Al_2_O_3_ vs. YAG for a given at.% dopant. At *c* = 0.25 at.%, *c*_vol_ = 1.18 × 10^20^ atoms/cm^3^ for Nd:Al_2_O_3_, compared to *c*_vol_ = 9.26 × 10^19^ atoms/cm^3^ for Nd:YAG, which is an increase of ~26%. Ultimately, this indicates that a 0.25 at.% Nd:Al_2_O_3_ ceramic will contain a suitable concentration of RE for lasing.

To obtain gain in an Nd:Al_2_O_3_ bulk polycrystalline material, processing techniques that will produce fully dense ceramics with fine average grain size (AGS) and/or that offer processing “windows” with increased rare-earth solubility are needed. Fortunately, the Nd solubility can be increased using high heating and cooling rates (to be discussed below), easing the necessity for extremely fine grains. Using a solid-state powder processing route along with a one-step simultaneous reaction/densification approach with CAPAD, we can achieve an Nd concentration as high as 0.35 at.% (Nd:Al ratio).

**Fig. 2:**
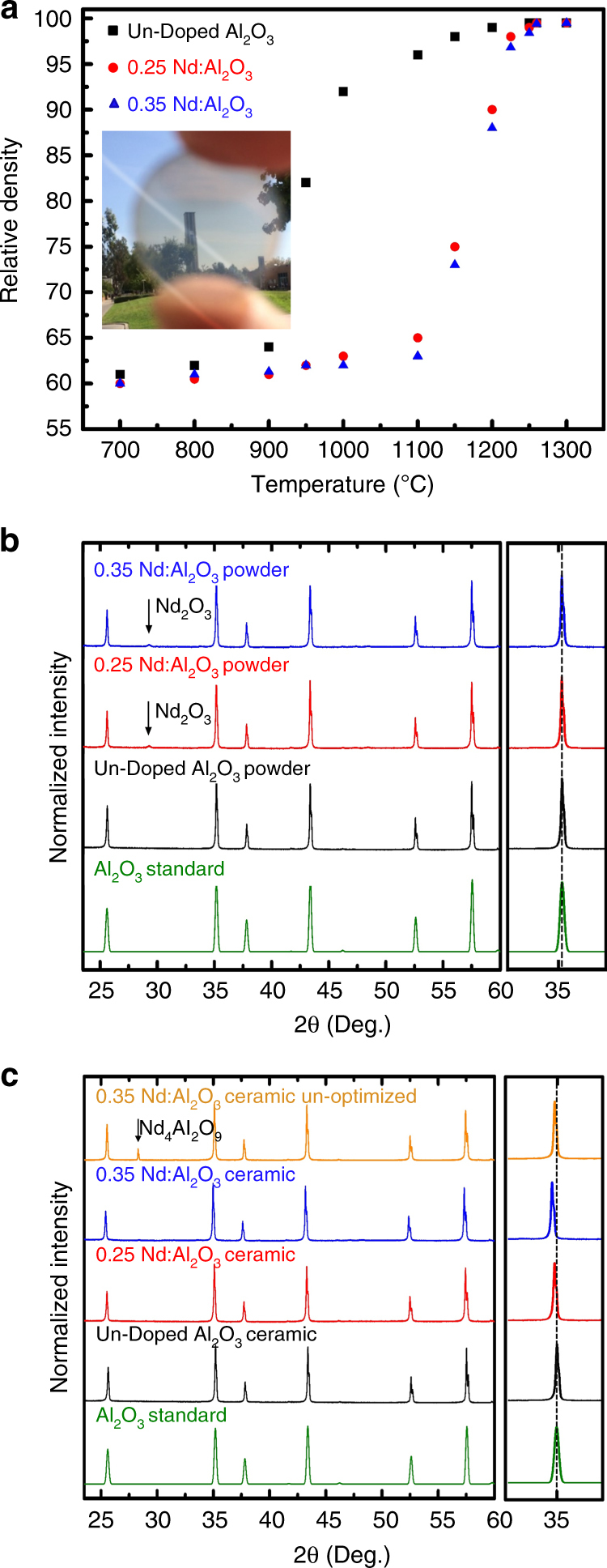
Physical and microstructural characterization of Nd:Al_2_O_3_. **a** the effect of CAPAD temperature on the relative density of un-doped and samples doped with 0.25 and 0.35 at.% Nd. The inset is a picture demonstrating long range transparency. **b** XRD profiles of the starting Al_2_O_3_ and Nd-doped Al_2_O_3_ powders. For the 0.25 and 0.35at. % powders, there are peaks attributed to the Nd_2_O_3_ dopant as indicated by arrows. **c** XRD profiles of Al_2_O_3_ and Nd-doped ceramics. The un-optimized Nd-doped sample shows a clear secondary phase (indicated with an arrow). The optimized samples do not show signs of a secondary phase present. The inset on the right clearly shows a peak shift relative to an *α*-Al_2_O_3_ standard (dashed line) for optimized Nd:Al_2_O_3_

At processing temperatures of 1200 °C (un-doped) and 1260 °C (Nd-doped), the samples have a fine AGS of ~250 nm, near the theoretical density, and are phase pure. As such, they possess long-range transparency (Fig. 2a) and when doped emit light at the characteristic Nd^3+^ wavelength of 1064 nm when pumped with 806 nm, which are prerequisites for gain. However, all samples processed at 1300 °C are diffuse and white due to an increased AGS to ~2.1 µm ± 0.25 µm for the un-doped α-Al_2_O_3_ and 1.9 µm ± 0.22 µm and 1.87 µm ± 0.23 µm for 0.25 at.% and 0.35 at.% Nd:Al_2_O_3_, respectively. At these larger grain sizes, the scattering efficiency is significantly higher (Fig. 1a).

The CAPAD processing parameters were varied to optimize the microstructure and properties of various concentrations of Nd:Al_2_O_3_ (see the Materials and methods for details). Figure 2a shows the effect of CAPAD temperature on the relative density of un-doped samples and others doped with 0.25 and 0.35 at.% Nd. The results show a sigmoidal temperature dependence, where the density increases abruptly at a temperature referred to as the densification on-set temperature, *T*_OD_. There is a clear influence of Nd dopant on *T*_OD_. For the Nd-doped Al_2_O_3_ samples, *T*_OD_ is ~200 °C higher than in the un-doped case (a shift from ~900°C to ~1100°C). There is also a small effect between the two different Nd concentrations on *T*_OD_. The densities of the 0.25 at.% Nd samples are slightly higher than those for the 0.35 at.% Nd samples at most processing temperatures. Nd addition also affects the temperature required to obtain full density; relative densities > 99% are achieved in the un-doped Al_2_O_3_ case at 1200 °C and ~1260 °C for the Nd:Al_2_O_3_ samples.

We have previously observed reduced densification kinetics caused by RE addition in reaction/densification of ceramics^19, 43^. This is due primarily to the presence of the RE oxide dopant powder along the particle/grain boundaries when the two phases are still separate reactants. In our previous work on alumina with Tb as a dopant, the decrease in density was lower compared to the present case of Nd at similar global concentrations^19^. The difference in behavior between the Nd and Tb dopants can be attributed to the larger ionic radius of Nd^3+^ (0.983 Å) compared to Tb^3+^ (0.923 Å). A similar shift in the *T*_OD_ with respect to RE ionic radius was reported for a Nd^3+^, Eu^3+^, and Er^3+^ doped Al_2_O_3_ system (0.2 at.% RE to Al_2_O_3_ ratio, ~0.04 at.% RE:Al) via free-sintering and hot-pressing by Drdlík et al.^44^. It is worth noting that in their work, the *T*_OD_ was significantly higher (>1400 °C), and a lower ~98% relative density was achieved at processing temperatures >1500 °C. The higher processing temperatures resulted in larger AGS (>500 nm) which diminished the material transmission and dopant concentration.

Figure 2b shows X-ray diffraction (XRD) profiles of the Al_2_O_3_ and Al_2_O_3_ + Nd_2_O_3_ powders after planetary ball milling (PBM) with varying Nd concentrations. These XRD spectra reveal a peak at 2*θ* = 30.72°, corresponding to the highest intensity peak for Nd_2_O_3_. Comparison of the XRD of the PBM starting powders to the α-Al_2_O_3_ reference does not show discernible peak shifts irrespective of Nd concentration, suggesting that Nd^3+^ doping into the α-Al_2_O_3_ matrix did not occur through mechanical alloying during PBM. This is expected considering the relatively low energy of the PBM conditions.

Figure 2c shows XRD spectra of fully dense polycrystals using optimized and non-optimized CAPAD conditions. The heating rates, processing temperatures, and hold times of the optimized and non-optimized cases were similar (HR = 300 °C min^−1^, *T* = 1260 °C, and HT = 5 min); the largest difference in each case was in the cooling rate, CR, which was significantly higher for the optimized case (Optimized CR = 300 °C min^−1^ and Non-optimized CR~42 °C min^−1^). The XRD spectra of the non-optimized sample reveal an unwanted secondary phase, Nd_4_Al_2_O_9_, (marked with an arrow). The highest intensity alumina peak is also at the same angle compared to the un-doped alumina ceramic, suggesting that Nd had not been adequately incorporated in the lattice.

By contrast, XRD of the ceramics processed using optimized CAPAD conditions reveal single phase α-Al_2_O_3_ with no signal from the starting Nd_2_O_3_ or from the ternary Nd_4_Al_2_O_9_ and NdAlO_3_ phases. This is in contrast to some previous reports that showed secondary phases in RE-doped α-Al_2_O_3_ that have been produced at RE concentrations above the equilibrium solubility limit with other processing approaches^45, 46^. Moreover, the XRD spectra of the optimized Nd-doped samples reveal clear peak shifts to lower angles with increasing Nd concentration. The dashed line in the inset on the right is the location of highest intensity peak from the reference. This shift is evidence of stretching of the α-Al_2_O_3_ lattice from the doping of Nd ions caused by CAPAD processing. The absence of the Nd_2_O_3_ reactant and ternary phases strongly indicates a fundamental difference in the reaction kinetics associated with CAPAD processing in comparison to that for traditional processing approaches.

We attribute the ability to incorporate high concentrations of RE into Al_2_O_3_ to the high heating and cooling rates we employed in CAPAD. The high heating rate ~300 °C min^−1^ allows us to reach the desired temperature quickly, minimizing unwanted grain growth^19, 47^ while achieving a near theoretical relative density, which are pre-requisites for high optical transparency in Al_2_O_3_. We previously observed an increase in reaction kinetics associated with high heating rates in the Ce:YAG system^43^. We found ~20-fold increases in reaction coefficients in comparison to reaction/densification in free-sintering using much slower heating rates. Since the largest difference between the optimized and un-optimized samples in this work was in the CR, we believe this parameter also plays a crucial role in RE incorporation. The Nd solubility increases at higher temperatures so that the high CR has the effect of “freezing in” Nd, thus minimizing segregation. There is a synergistic effect between fine AGS and RE incorporation during CAPAD. A more detailed investigation of the relationships between CR, microstructure, and optical properties is underway but is beyond the scope of this communication.

**Fig. 3:**
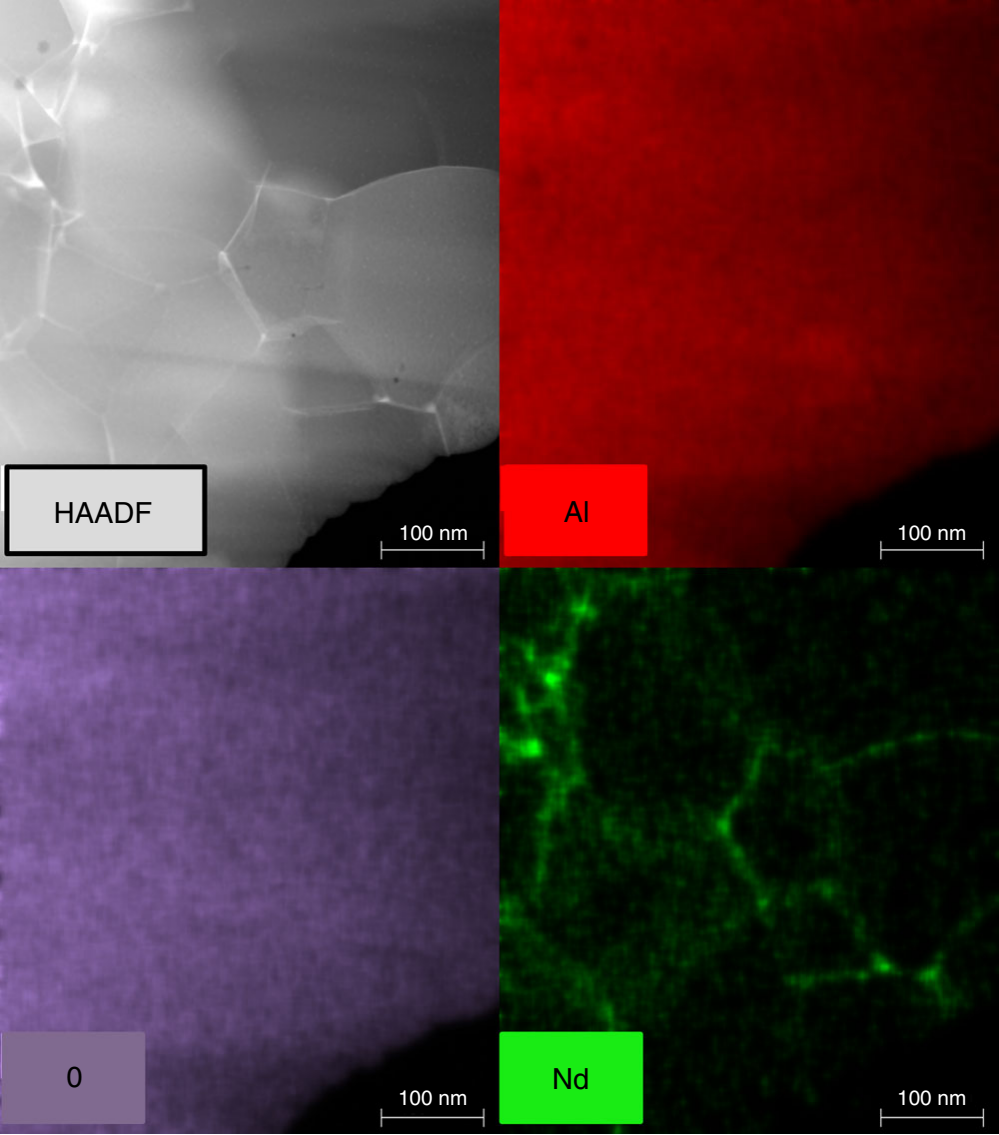
High-angle annular dark-field transmission (HAADF) TEM micrograph of 0.35 at.% Nd:Al_2_O_3_ bulk ceramic (optimized sample) with corresponding energy-dispersive X-ray spectroscopy (EDS) elemental maps for Al, O, and Nd (L-Lines). The EDS maps reveal that a significant portion of the Nd dopant is found within the matrix. In addition, there is some Nd along some grain boundaries and triple points

We used TEM to further confirm incorporation of Nd into the alumina matrix. A high-angle annular dark-field (HAADF) TEM micrograph and corresponding energy-dispersive X-ray spectroscopy (EDS) distribution maps of a 0.35 at.% Nd:Al_2_O_3_ polycrystal (*T* = 1260°C, HT = 5 min, HR = 300 °Cmin^−1^, and CR = 300 °Cmin^−1^) are shown in Fig. 3a. The EDS maps reveal that a significant portion of the Nd dopant is found within the matrix and along some grain boundaries and triple points. The minimal segregation corroborates the XRD spectra in Fig. 2c, which shows a shift in the XRD peaks to lower 2*θ* angles and does not show the presence of unwanted secondary phases. This is in-line with observations by Rohrer, Harmer and co-workers ^48, 49^ showing differences in the local grain boundary structure in RE-doped α-Al_2_O_3_ and an increasing concentration gradient from the grain interior towards the grain boundary.

**Fig. 4:**
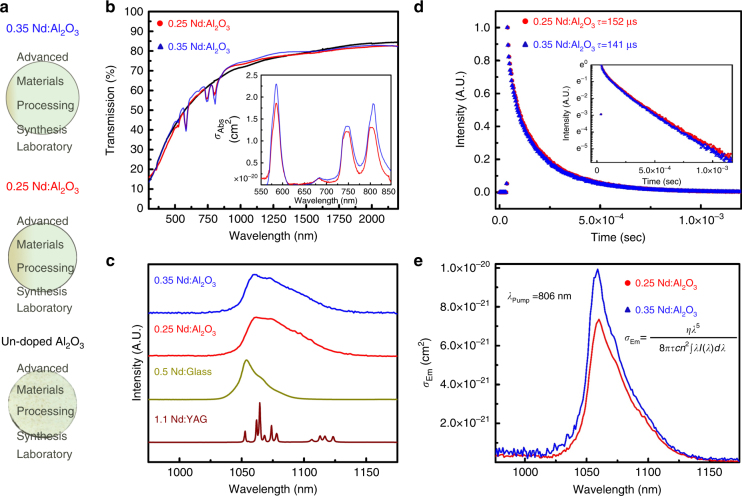
Optical properties of Nd:Al_2_O_3_. **a** Pictures of Nd-doped and undoped ceramics. **b** Transmission measurements of the Nd:Al_2_O_3_ and undoped Al_2_O_3_. All the ceramics show high transmission, and importantly, the Nd-doped samples have absorption bands characteristic of Nd^3+^ transmission. The corresponding absorption cross sections in the area of interest are shown in the inset. **c** PL emission spectra for the 0.25 at.% and 0.35 at.% Nd^3+^:Al_2_O_3_ samples along with 0.5 at.% Nd^3+^:Glass and 1.1 at.% Nd^3+^:YAG single crystal. The pump source is an 806 nm laser diode. The PL reveal broadened lines attributed to the ^4^F_3/2_ → ^4^I_11/2_ electronic transitions. **d** the radiative lifetimes at 1064 nm for the Nd:Al_2_O_3_ ceramics produced under similar CAPAD processing conditions, and log scale intensity is also shown. The lifetimes are 152 μs and 141 μs for the 0.25 and 0.35 at.% Nd:Al_2_O_3_, respectively. **e** the resultant emission cross-sections, *σ*_Em_, using the Fuchtbauer–Landenburg relationship (Eq. 2). The emission cross section peak is *σ*_Em_ = 7.5 × 10^−21^ cm^2^ for 0.25 at.% and 9.8 × 10^−21^ cm^2^ for 0.35 at.% Nd:Al_2_O_3_ ceramics

The optical transparencies of the consolidated bulk Nd:Al_2_O_3_ polycrystals are shown in Fig. 4a with the corresponding transmission spectra presented in Fig. 4b. The transmission values of our undoped alumina ceramics rival those previously reported for sinter-HIPed samples^38^ and high pressure CAPAD^50^. More importantly, the Nd-doped samples have similar transmissions. In the area of interest for lasing of Nd^3+^ media at ~1064 nm (^4^F_3/2_ → ^4^I_11/2_ transition), the transmission is ~75% for the Nd:Al_2_O_3_. We attribute this high transmission to the high density (>99%), fine AGS (~250 nm), low Nd segregation, and lack of secondary (undesired) phases in the Nd:Al_2_O_3_. It is important to note that this transmission is not corrected for refection losses. When corrected for reflection losses, the transmission at 1064 nm is ~90%, leading to a loss coefficient (absorption+scattering) of ~1.317 cm^−1^. For laser oscillation, a gain greater to this total loss is required for net positive gain. Our single-pass gain measurements presented below show that the optical quality of our ceramics is indeed suitable for lasing.

## Discussion

One remarkable difference in the Nd:Al_2_O_3_ transmission spectra is the presence of the absorption bands centered at *λ* = 583 nm (2.12 eV), 745 nm (1.85 eV), and 806 nm (1.54 eV), which correspond to the ^4^G_5/2_, ^4^F_7/2_, and ^4^F_5/2_ Stark transitions from the ^4^I_9/2_ manifold^51, 52^. We believe this is the first time that absorption bands associated with RE doping have been observed in Al_2_O_3_ transmission spectra and strongly evidence that the Nd^3+^ dopant is optically active within the ceramic matrix^53^. The center of the Nd^3+^ absorption bands in Al_2_O_3_ are slightly blue shifted (~2.5 nm) in comparison with that for Nd:YAG single crystals^51, 52^. The absorption bands are broadened in Nd:Al_2_O_3_ to Δ*λ*~23 nm (FWHM) from ~Δ*λ*~2 nm compared to Nd:YAG^53^, which is consistent with our observations that the Nd^3+^ is found on multiple doping sites within the alumina matrix. Moreover, the depth of the absorption bands increases with dopant concentration, indicating greater optical activity from the Nd^3+^ ions within the 0.35 at.% Nd:Al_2_O_3_ sample.

The absorption cross-sections *σ*_abs_ for the region of interest are shown in the inset in Fig. 4b. These *σ*_abs_ were calculated from the measured transmissions corrected for reflection and scattering losses^39^. In dense polycrystalline ceramics with anisotropic crystal structure (uniaxial in this case), one should correct for scattering losses caused by the birefringence to not overestimate *σ*_abs_. We corrected for scattering losses using the Rayleigh–Gans–Debye (RGD) approach in which the scattering has a 1/*λ*^2^ dependence, as discussed previously for transition metal-doped alumina^39^. The excellent agreement between the calculated and measured transmission spectra (not shown here) for the un-doped Al_2_O_3_ ceramics confirm that the uniaxial crystal structure is the main source of scattering as opposed to porosity and validates the use of the correction method.

For the ^4^F_5/2_ transition, which is of interest for diode-pumped lasers, the peak *σ*_abs_ are 1.36 × 10^−20^ cm^2^ and 1.69 × 10^−20^ cm^2^ for the 0.25 at.% and 0.35 at.% Nd:Al_2_O_3_, respectively. These cross-sections compare well with single-crystal 1.1 at.% Nd:YAG, *(σ*_abs_~ 7.7 × 10^−20^ cm^2^). The slightly lower *σ*_abs_ in Nd:Al_2_O_3_ may be caused by Nd sites that are not optically active or absorption band broadening, which also occurs in Nd:Glass and Nd:YVO_4_^54, 55^.

Figure 4c presents the PL emission spectra for the 0.25 at.% and 0.35 at.% Nd^3+^:Al_2_O_3_ ceramics, 0.5 at.% Nd^3+^:Glass (Schott), and 1.1 at.% Nd^3+^:YAG (single crystal, Litton Technologies, Inc.) resulting from pumping at *λ* = 806 nm. All the media show emission at similar wavelengths but different line shapes and bandwidths for the ^4^F_3/2_ → ^4^I_11/2_ transition. The single-crystal profile shows narrow, well-defined peaks typical of single site doping. By contrast, emission peaks in Nd^3+^:Al_2_O_3_ appear to be inhomogeneously broadened, similar to that for Nd^3+^:Glass, although the overall PL bandwidth is wider than for the laser glass. Inhomogeneous broadening of the Nd^3+^:Al_2_O_3_ emission lines is not surprising given that Nd ions are found on multiple sites, including at grain interiors, grain boundaries and triple points (Fig. 3). This broadening contrasts with PL behavior reported by Waeselmann in 2 at.% Nd:Al_2_O_3_ on thin films produced with PLD. These authors demonstrated lasing in epitaxial films that showed narrow emission lines for the ^4^F_3/2_ → ^4^I_11/2_ transition, producing PL at 1097 nm^35^. The shifted emission peak compared to our results and single-crystal Nd:YAG is not surprising because epitaxial thin films often display shifts compared to bulk materials. The authors attribute the sharp emission peaks to single site doping, in particular the substitution of Nd^3+^ onto the Al^3+^ lattice. Despite the sharp PL peaks, they did not observe a significant absorption cross-section, which they attribute to the possibility of dead Nd sites, which do not contribute to absorption or PL.

The gain bandwidth (*G*_bw_) can be approximated by measuring the full-width at half-maximum (FWHM) of the PL emission peaks. We obtain *G*_bw_ = 0.6 nm (0.16 THz) for Nd^3+^:YAG and *G*_bw_ = 20 nm (5.4 THz) for Nd^3+^:Glass, which agree well with previous measurements^53, 55^. Remarkably, the *G*_bw_ are *~*49 nm (13 THz) which we believe are the highest bandwidths measured for Nd^3+^ in any media. For bandwidth-limited pulses, the achievable pulse duration of a gain medium is determined by *G*_bw_. The broader the emission bandwidth, the shorter the pulse; the pulse width can be estimated using ∆*τ*_P_ *=* 1/*G*_bw_. Using *G*_bw_ measurements, we find ∆*τ*_P_~7.7 fs. The large bandwidth of Nd^3+^:Al_2_O_3_ promises the generation of high peak-power lasers by generating ultra-short time pulses. These bandwidth-limited pulse widths represent a 2.5-fold increase in the single-shot peak power over Nd^3+^:Glass and >80-fold increase over Nd^3+^:YAG (∆*τ*_P_ = 6.3 ps for Nd^3+^:YAG and ∆*τ*_P_ = 18.5 fs for Nd^3+^:Glass) through pulse width compression. These estimated improvements are conservative because thermal shock resistance for Nd:Al_2_O_3_ (*R*_s_~19,500 Wm^−1^) is superior to Nd:YAG (*R*_s_~800 Wm^−1^) and Nd:Glass (*R*_s_~1 Wm^−1^), indicating the possibility of scaling peak-power extraction accordingly.

Given these interesting absorption and PL characteristics, we measured the radiative lifetimes, *τ*, at 1064 nm for the Nd:Al_2_O_3_ ceramics. The lifetimes are 152 μs and 141 μs for the 0.25 and 0.35 at.% Nd:Al_2_O_3_, respectively (Fig. 4d). These lifetimes compare well with those of other proven gain media; they are longer than those observed by Waeselmann in 2 at.% Nd:Sapphire but are shorter than those of Nd:YAG (230 μs^54^) and Nd:Glass (330 μs^24^). The small decrease in *τ* as the Nd concentration increases for the 0.25 to the 0.35 at.% samples may indicate the onset of concentration quenching. By contrast, the un-optimized 0.35 at.% Nd:Al_2_O_3_ sample results in a significant decrease in *τ*~50 μs. This is not surprising because we observed clear secondary phases in the XRD analysis. Further spectroscopic and processing studies are required to fully understand concentration quenching in Nd:Al_2_O_3_.

From the PL emission spectra, we determined the emission cross-sections *σ*_Em_ using the Fuchtbauer–Landenburg relationship^56^,

2 $$\sigma _{\rm{Em}} = \frac{{n\lambda ^5}}{{8\pi \tau cn^2\!\int\! I\left( \lambda \right){\rm{d}}\lambda }}$$

The *σ*_Em_ are large and adequate for lasing across the PL bandwidth; the peak *σ*_Em _= 7.5 × 10^−21^ cm^2^ for 0.25 at% and 9.8 × 10^−21^ cm^2^ for 0.35 at.% optimized ceramics. These *σ*_Em_ are consistent with *σ*_Abs_ derived from the measured transmission spectra. By contrast, σ_Em_ is 3.1 × 10^−22^ cm^2^ for the un-optimized sample. The substantially lower *σ*_Em_ proves that the presence of second phases deteriorates the optical activity for the Nd-dopant.

**Fig. 5:**
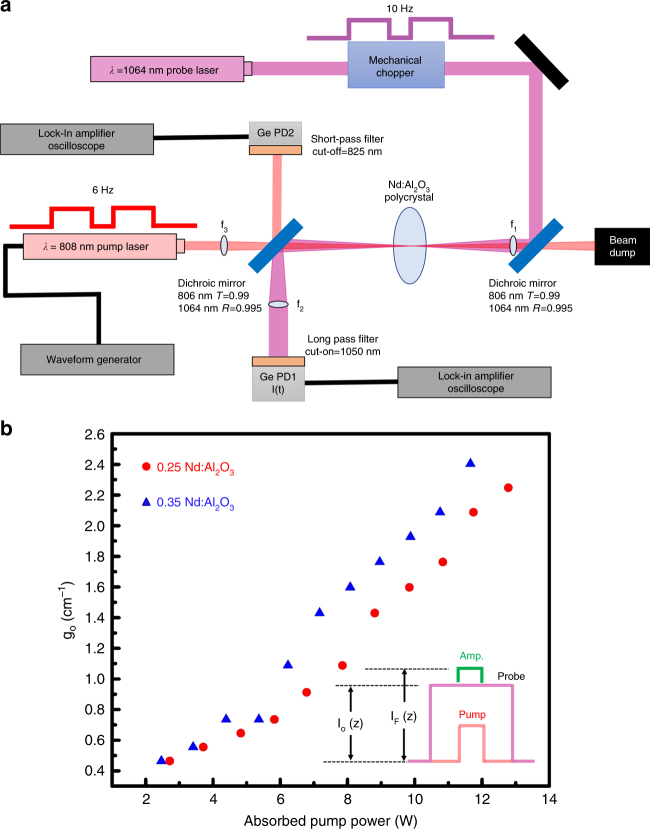
Demonstration of optical gain. **a** Schematic of the single-pass measurement set-up. **b** Single-pass gain coefficients of the 0.25 at.% and 0.35 at.% Nd^3+^:Al_2_O_3_ bulk polycrystalline ceramics. The inset schematically shows the relationship between the pump, probe and gain signals and Eq. 3

To unambiguously ascertain the viability for lasing in Nd^3+^:Al_2_O_3_, we measured their small-signal gain coefficients using a single pass arrangement similar to one used by Lai^57^. The schematic for the optical arrangement is shown in Fig. 5a. Briefly, a 1064 nm probe beam was passed through a specimen at a constant incident power. An 806 nm pump laser was introduced onto the same spatial location on the test specimens using a dichroic optic with high-transmission (99% at 806 nm) and high-reflection (99.5% at 1064 nm). The increase/decrease in the probe beam intensity as a function of absorbed pump power was monitored by the same photodiode. We used a modified version of the Beer–Lambert law for homogenous/Doppler broadened gain media to measure gain coefficients:

3 $$I_{\rm{F}}\left( z \right) = I_{\rm{o}}(z)e^{[g_{\rm{o}}] \cdot z}$$

where *I*_o_*(z)* and *I*_F_*(z)* are the intensities of the probe laser after having passed through the test specimen of thickness *z*, prior to and with pumping, respectively, and *g*_0_ is the small-signal gain coefficient, obtained here in a single-pass arrangement.

Figure 5b plots the gain coefficients for the 0.25 at.% and 0.35 at.% Nd^3+^:Al_2_O_3_ ceramics as a function of absorbed pump power. We observe a gain in the transmitted probe laser at absorbed pump powers >2.25 W for both materials. The magnitude of *g*_0_ increases approximately linearly as a function of the absorbed pump power, and in this power range, we do not observe gain saturation. The gain values are as high as 2.27 cm^−1^ and 2.42 cm^−1^ for the 0.25 at.% and 0.35 at.% Nd^3+^ concentrations, respectively. These small-signal gain coefficients compare well to values for Nd:YAG (2 cm^−1^)^58^, Nd:Glass (5 cm^−1^)^54^, Ti:Sapphire (1 cm^−1^)^58^, and Cr:Sapphire (1 cm^−1^)^58^. As discussed above, our materials have scattering and absorption losses that are ~1.317 cm^−1^ after having corrected for reflection loss. It is worth noting that reflection loss can be mitigated using anti-reflection coatings on the ceramic. These single-pass gain measurements reveal a net positive gain at absorbed pump powers of >8 W and 7.2 W for the 0.25 at.% and 0.35 at.% Nd:Al_2_O_3_, respectively, where *g*_0_ surpasses the absorption and scattering loss. These measurements explicitly show that the optical quality (transparency, *τ*, *σ*_Abs,_ and *σ*_Em_) of Nd^3+^:Al_2_O_3_ bulk ceramics is suitable for amplification and oscillation should optical feedback be introduced, i.e., within a laser cavity employing AR coatings on the gain medium.

We attribute the demonstration of gain to the unique nanostructure of the ceramics. The fine AGS results in an Al_2_O_3_ with a large grain boundary volume, which facilitates the accommodation of the RE without significant concentration quenching. In addition to microstructural control, high heating and cooling rates during CAPAD processing also affect the incorporation of Nd^3+^ into the grain and grain boundary regions without the formation of unwanted secondary phases that lead to poor optical activity.

In summary, we introduce a powder processing route in conjunction with single-step CAPAD reaction/densification to produce transparent bulk polycrystalline Nd^3+^:Al_2_O_3_ with Nd incorporated at concentrations of 0.25 at.% and 0.35 at.%. The ceramics have a high transmission at 1064 nm and display absorption bands at *λ* = 585 nm, 748 nm, and 806 nm, corresponding to transitions from the ^4^I_9/2_ manifold of optically active Nd^3+^ that result in high peak absorption cross-sections. The PL bandwidth of ~13 THz centered at 1064 nm represents a new record for Nd^3+^ media, thus permitting the generation of ultra-short pulses. The radiative lifetimes are long and yield a large emission cross-section, which result in an optical gain that is suitable for amplification and lasing. Moreover, the significantly higher *R*_S_~19,500 W/m of Nd^3+^:Al_2_O_3_ promises a significantly higher duty-cycle and/or peak-power, making Nd^3+^:Al_2_O_3_ a potentially revolutionary gain material. Finally, we note that the nano/microstructural strategies demonstrated here should be applicable to many other oxide and nitride gain systems that were not previously believed to be applicable as laser ceramics and thus represents a new approach to producing gain media.

## Materials and methods

### Relations between interionic distance, grain size, and effective length

An important factor for gain is the average distance between dopant ions, $\tilde l$. Dopant concentrations *c* are usually reported in [at.%] relative to cations. It is convenient to think about interionic distances using volumetric concentration *c*_vol_ [ions/cm^3^] because $\tilde l$ scales with the total number of ions in a volume *V* such that $\tilde l \propto \root {3} \of {{1/c_{\rm{vol}}V}}$. Although calculations or measurements of $\tilde l$ can be complicated, it is easy to obtain a good estimate of ~*l* using a regular pattern of dopants such as a simple cubic cell with RE on each corner with *l* as a cell length. In this case, $\tilde l\sim l = \root {3} \of {{1/c_{\rm{vol}}V}}$. We consider laser quality Nd:YAG as an example, where the typical dopant concentration is 0.5–2at.%. In the *c* *=* 2 at.% case, *c*_vol_ = 7.53 × 10^20^ ions/cm^3^ such that $\tilde l$ ~ 1.09 nm.

It is interesting to consider alternate dopant distributions. Consider one grain of gain media approximated as a cube with a global volumetric dopant concentration *c*_vol_ [ions/cm^3^]. The total number of ions *N* in the volume of that cube is equal to *c*_vol_*d* ^3^, where *d* is the cube edge length. If all the dopant ions in that cube are placed on the surface (i.e., grain boundary) rather than in the grain volume, one can calculate the effective length (edge length) *d*_eff_ necessary to accommodate all the dopants for a given arrangement on the surface of the cube. For simplicity, we can approximate the random arrangement of ions as a regular square unit cell with cell parameter 2*r* + *l*, where *r* is ionic radius, and *l* is the distance between dopant ions. Because there are 6 sides to a cube, *d*_eff_ as a function of grain size (edge length) *d* is

4 $$d_{\rm{eff}} = \sqrt {\frac{{d^3c_{\rm{vol}}(2{r} + {l})^2}}{6}}$$

A value of *r* = 1.15 Å for Nd ions and *l* = 1 nm was used for calculations because 1 nm is a good approximation of $\tilde l$, as shown above.

### Powder preparation

Commercially available α-Al_2_O_3_ (99.99% purity, Taimei Chemicals, Japan) was processed as received (un-doped) and doped with Nd_2_O_3_ (99.99% purity, Alfa Aesar, USA). The powders were mixed to achieve doping levels (Nd^3+^:Al^3+^) of 0.25 and 0.35 at.%. The powders were mixed dry in an alumina mortar by hand for 20 min, which was followed by low-energy ball milling for 12 h with ultra-high purity (UHP, 99.99% purity) water as a dispersant. The slurries were sieved and centrifuged for 15 min at 3400 RPM. The powders were dried in a vacuum oven at 70 °C under a vacuum of 30 mm Hg for 12 h. Dried powders were subsequently planetary ball milled with UHP water at 150 RPM for 6 h. Finally, the powders were sieved and dried in air at 120 °C for 12 h and kept dry until consolidation.

### CAPAD processing

The powders were densified by CAPAD^47^ using a graphite die (19 mm outer and 10 mm inner diameter). This die and plunger set was secured between two 19 mm punches and placed within a larger graphite die with a 19 mm inner diameter. The die and powder set were placed into the CAPAD, and a vacuum of 10^−3^ Torr was established. The powders were pre-pressed at 106 MPa for 20 min, after which the load was released. An ultimate pressure of 106 MPa with a pressure ramp of 35.33 MPamin^−1^ was applied and held constant. In parallel with the application of pressure, the samples were subjected to a heating rate of ~300 °Cmin^−1^ and a maximum temperature ranging between 700 and 1300 °C with a hold time of 5 min. The temperature was monitored with a dual wavelength optical pyrometer focused at the die midpoint.

### Microstructural characterization

The powders and densified ceramics were characterized using XRD using Cu Kα_1_ (*λ* = 1.54058 Å) radiation on a PANalytical Empyrean Diffractometer (PANalytical, Almelo, The Netherlands) using a step size of 2*θ* = 0.005°. Published standards were used for comparison: Nd_2_O_3_ (ICSD#26867) and α-Al_2_O_3_ (ICSD#:63647).

The AGS of the densified ceramics were obtained from fracture surfaces by measuring >300 grains in multiple micrographs at random locations. The fractured surface was sputter coated with a thin film of Pt/Pd before examination with a Phillips XL30 field emission scanning electron microscope. EDS mapping was performed using a Titan Themis 399 Scanning-TEM (STEM). The TEM specimen was prepared using a gallium focused ion beam (FIB) and attached to a copper TEM grid using a Pt FIB.

### Transmission and photoluminescence measurements

The samples were polished with diamond suspensions to 0.5 µm. The final specimen thickness was 0.8 mm ± 0.05 mm. Transmission spectra were taken on a Varian Cary 500 UV-VIS-IR spectrometer from 300 nm to 2200 nm at normal incidence in single-beam mode with a rectangular spot size of 2 mm by 9 mm, using a scan rate of 0.2 nm s^−1^.

PL was measured on a Horiba Spex Fluorolog 3 Spectrophotometer using an 806 nm laser diode as the excitation source with a 100 mW incident power and a spot size of 2 mm. Measurements were taken in front face mode at a 45° angle of incidence (AOI) on polished samples. Emission scans were taken between *λ* = 1000 nm and *λ* = 1100 nm with an integration time of 1 snm^−1^.

### Photoluminescence lifetime measurements

PL lifetimes (pump = 806 nm) were obtained using a pulsed tunable laser (Continuum Surelite with optical parametric oscillator). The pulse width was 6 ns, the spot size was 6 mm, and the incident energy was 3 mJ per pulse. The ceramics were mounted within a Horiba Spex Fluorolog 3 Spectrophotometer, which was coupled to a germanium photodiode and synchronized to a Tektronix TPS2024B oscilloscope. The monochromators were adjusted to observe 1064 nm, with a spectral bandwidth of 1 nm. An optical notch filter centered at 1064 nm with 8 nm FWHM transmission band was used to further isolate the pump source. Measurements were taken in front face mode at 45° AOI. A double-exponential was used to fit data and extract the lifetimes, where *τ* is defined as the time required for the intensity to decrease by 1/*e*^27^.

### Single-pass optical gain

Optical gain was measured using a single-pass arrangement similar to that of Lai et al.^57^, which is shown schematically in Fig. 5b. The samples were held within an aluminum mount atop a 6-axis kinematic mount that was modified for water cooling, allowing a constant sample temperature of 15 °C throughout the measurements.

A continuous wave Nd:YAG laser operating at the fundamental wavelength (*λ* = 1064 nm) was used as the probe laser. The collimated probe beam (~1 mm diameter) was focused onto the sample with a 100 mm focal length lens, resulting in a FWHM spot size of ~220 µm. A fiber coupled Coherent FAP 35 W laser diode (*λ* = 806 nm) and collimator composed the pumping source. The pump laser was focused onto the sample collinear to, but counter-propagating with respect to the probe using a 35 mm focal length lens, resulting in a spot size of ~400 µm. The spot sizes were determined by fitting a Gaussian profile to the probe laser and a top-hat profile to the pump laser from CCD images of the focused beams. The pump beam waist was injected into the arrangement via a dichroic mirror (Thorlabs DMSP1000) with a reflective cut-on wavelength of 1000 nm at a 45° AOI. In addition to the factory dielectric coatings, an additional anti-reflective coating for 806 nm was deposited onto the dichroic optics, which maximized the deliverable pump power onto the test specimens while minimizing stray Fresnel reflections for the pump laser.

The focusing optics for the probe and pump beams were mounted on six-axis kinematic fixtures, allowing a precise spatial alignment of the beams within a single sample interaction volume. The pump and probe beam power were monitored with germanium photodetectors (Thorlabs PDA50B) PD1 and PD2, respectively, which were optically isolated to the desired wavelengths with low and high-pass filters. The pump and probe lasers were operated in quasi-continuous mode using 8 Hz and 10 Hz boxcar waveforms, respectively. The fluctuations in the pump and probe laser intensities were recorded using a lock-in amplifier in parallel with an oscilloscope at their respective operating frequencies. This ensures that fluctuations in PD signals are isolated. The photodetectors were calibrated against an optical power meter (Ophir Nova 2).

## References

[1] Wieg AT, Kodera Y, Wang Z, Dames C, Garay JE (2015). Thermomechanical properties of rare-earth-doped AlN for laser gain media: the role of grain boundaries and grain size. Acta Mater..

[2] Kim W (2012). Ceramic windows and gain media for high-energy lasers. Opt. Eng..

[3] Kerse C (2016). Ablation-cooled material removal with ultrafast bursts of pulses. Nature.

[4] Liu RM (2017). Strong light-matter interactions in single open plasmonic nanocavities at the quantum optics limit. Phys. Rev. Lett..

[5] Popmintchev T (2009). Phase matching of high harmonic generation in the soft and hard X-ray regions of the spectrum. Proc. Natl Acad. Sci. USA.

[6] Di Piazza A, Müller C, Hatsagortsyan KZ, Keitel CH (2012). Extremely high-intensity laser interactions with fundamental quantum systems. Rev. Mod. Phys..

[7] Steinmeyer JD (2010). Construction of a femtosecond laser microsurgery system. Nat. Protoc..

[8] Polini M (2016). Tuning terahertz lasers via graphene plasmons. Science.

[9] Ikesue A, Aung YL (2006). Synthesis and performance of advanced ceramic lasers. J. Am. Ceram. Soc..

[10] Waxler RM, Cleek GW, Malitson IH, Dodge MJ, Hahn TA (1971). Optical and mechanical properties of some neodymium-doped laser glasses. J. Res. Natl Bur. Stand A.

[11] Klein PH, Croft WJ (1967). Thermal conductivity, diffusivity, and expansion of Y2_O_3, Y3_A_l_5O_1_2,_ and LaF3 in the range 77°–300°K. J. Appl. Phys..

[12] Ikesue A, Aung YL (2008). Ceramic laser materials. Nat. Photonics.

[13] Ikesue A, Aung YL, Taira T, Kamimura T, Yoshida K (2006). Progress in ceramic lasers. Annu. Rev. Mater. Res..

[14] Ikesue A (2002). Polycrystalline Nd:YAG ceramics lasers. Opt. Mater..

[15] Xu CW, Yang CD, Zhu HY, Ye YL, Duan YM (2017). Diode-pumped Nd:LuAG ceramic laser on 4^F^3_/2-_4^I^1_3/2_ transition. Opt. Mater..

[16] Fornasiero L, Mix E, Peters V, Petermann K, Huber G (2000). Czochralski growth and laser parameters of RE^3+^-doped Y_2_O_3_ and Sc_2_O_3_. Ceram. Int.

[17] Choudhary A, Beecher SJ, Dhingra S, D’Urso B, Parsonage TL (2015). 456-mW graphene Q-switched Yb:yttria waveguide laser by evanescent-field interaction. Opt. Lett..

[18] Toci G, Vannini M, Ciofini M, Lapucci A, Pirri A (2015). Nd^3+^-doped Lu_2_O_3_ transparent sesquioxide ceramics elaborated by the spark plasma sintering (SPS) method. Part 2: first laser output results and comparison with Nd^3+^-doped Lu_2_O_3_ and Nd^3+^-Y_2_O_3_ ceramics elaborated by a conventional method. Opt. Mater..

[19] Penilla EH, Kodera Y, Garay JE (2013). Blue-green emission in terbium-doped alumina (Tb: Al_2_O_3_) transparent ceramics. Adv. Funct. Mater..

[20] Lupei V, Lupei A, Ikesue A (2008). Transparent polycrystalline ceramic laser materials. Opt. Mater..

[21] Powell RW, Ho CY, Liley PE (1966). Thermal Conductivity of Selected Materials. National Standard Reference Data Series.

[22] Yao WL, Liu J, Holland TB, Huang L, Xiong YH (2011). Grain size dependence of fracture toughness for fine grained alumina. Scr. Mater..

[23] Li W. W., He D. B., Li S. G., Chen W., Chen S. B. et al. Optical and thermal properties of a new ND-doped phosphate laser glass. In *Proc. SPIE Pacific Rim Laser Damage 2013: Optical Materials for High Power Lasers*. 878629 (SPIE, Shanghai, China, 2013).

[24] Koechner, W. *Solid-State Laser Engineering* (Springer, Berlin, 2006).

[25] Maiman TH (1960). Stimulated optical radiation in ruby. Nature.

[26] Wall KF, Sanchez A (1990). Titanium sapphire lasers. Linc. Lab J..

[27] Chambers MD, Clarke DR (2009). Doped oxides for high-temperature luminescence and lifetime thermometry. Annu Rev. Mater. Res.

[28] Williams GR, Bayram SB, Rand SC, Hinklin T, Laine RM (2001). Laser action in strongly scattering rare-earth-metal-doped dielectric nanophosphors. Phys. Rev. A.

[29] Li B, Williams G, Rand SC, Hinklin T, Laine RM (2002). Continuous-wave ultraviolet laser action in strongly scattering Nd-doped Alumina. Opt. Lett..

[30] Song Q, Li CR, Li JY, Ding WY, Li SF (2006). Photoluminescence properties of the Yb: Er co-doped Al_2_O_3_ thin film fabricated by microwave ECR plasma source enhanced RF magnetron sputtering. Opt. Mater..

[31] Zhou B, Xiao ZS, Huang AP, Yan L, Zhu F (2008). Effect of Tm–Er concentration ratio on the photoluminescence of Er–Tm: Al_2_O_3_ thin films fabricated by pulsed laser deposition. Prog. Nat. Sci..

[32] Serna R, Nuñez-Sanchez S, Xu F, Afonso CN (2011). Enhanced photoluminescence of rare-earth doped films prepared by off-axis pulsed laser deposition. Appl. Surf. Sci..

[33] Kumaran R, Webster SE, Penson S, Li W, Tiedje T (2009). Epitaxial neodymium-doped sapphire films, a new active medium for waveguide lasers. Opt. Lett..

[34] Kumaran R, Tiedje T, Webster SE, Penson S, Li W (2010). Epitaxial Nd-doped α-(Al1_−_x_*G*_ax)2_O_3 films on sapphire for solid-state waveguide lasers. Opt. Lett..

[35] Waeselmann SH, Heinrich S, Kränkel C, Huber G (2016). Lasing of Nd^3+^ in sapphire. Laser Photonics Rev..

[36] Waeselmann S. H., Heinrich S., Kraenkel C., Huber G. Lasing in Nd^3+^-doped sapphire. Adv. Solid State Lasers. 6–8pp (OSA, Berlin, Germany, 2015).

[37] Waeselmann SH, Rüter CE, Kip D, Kränkel C, Huber G (2017). Nd: sapphire channel waveguide laser. Opt. Mater. Express.

[38] Apetz R, Van Bruggen MPB (2003). Transparent alumina: a light-scattering model. J. Am. Ceram. Soc..

[39] Penilla EH, Hardin CL, Kodera Y, Basun SA, Evans DR (2016). The role of scattering and absorption on the optical properties of birefringent polycrystalline ceramics: modeling and experiments on ruby (Cr: Al_2_O_3_). J. Appl. Phys..

[40] Krebs JK, Happek U (2001). Yb^3+^energy levels in a-Al_2_O_3_. J. Lumin..

[41] Sanamyan T, Pavlacka R, Gilde G, Dubinskii M (2013). Spectroscopic properties of Er^3+^-doped α-Al_2_O_3_. Opt. Mater..

[42] Pecharromán C, Mata-Osoro G, Díaz LA, Torrecillas R, Moya JS (2009). On the transparency of nanostructured alumina: Rayleigh-Gans model for anisotropic spheres. Opt. Express.

[43] Penilla EH, Kodera Y, Garay JE (2012). Simultaneous Synthesis and densification of transparent, photoluminescent polycrystalline YAG by current activated pressure assisted densification (CAPAD). Mater. Sci. Eng. B.

[44] Bodišová K, Klement R, Galusek D, Pouchlý V, Drdlík D (2016). Luminescent rare-earth-doped transparent alumina ceramics. J. Eur. Ceram. Soc..

[45] Thompson AM, Soni KK, Chan HM, Harmer MP, Williams DB (1997). Dopant distributions in rare-earth-doped alumina. J. Am. Ceram. Soc..

[46] Cho J, Wang CM, Chan HM, Rickman JM, Harmer MP (2002). A study of grain-boundary structure in rare-earth doped aluminas using an EBSD technique. J. Mater. Sci..

[47] Garay JE (2010). Current-activated, pressure-assisted densification of materials. Annu Rev. Mater. Res.

[48] Cantwell PR, Ma SL, Bojarski SA, Rohrer GS, Harmer MP (2016). Expanding time-temperature-transformation (TTT) diagrams to interfaces: a new approach for grain boundary engineering. Acta Mater..

[49] Bojarski SA, Stuer M, Zhao Z, Bowen P, Rohrer GS (2014). Influence of Y and La additions on grain growth and the grain-boundary character distribution of alumina. J. Am. Ceram. Soc..

[50] Grasso S, Yoshida H, Porwal H, Sakka Y, Reece M (2013). Highly transparent α-alumina obtained by low cost high pressure SPS. Ceram. Int..

[51] Yoon SJ, Mackenzie JI (2014). Cryogenically cooled 946nm Nd: YAG laser. Opt. Express.

[52] Krupke W (1971). Radiative transition probabilities within the 4f3 ground configuration of Nd: YAG. IEEE J. Quantum Electron.

[53] Kaminskii, A. A. *Laser Crystals: Their Physics and Properties*. (Springer, Berlin Heidelberg, 1990).

[54] Silfvast WT (2004). Laser Fundamentals..

[55] Campbell JH, Suratwala TI (2000). Nd-doped phosphate glasses for high-energy/high-peak-power lasers. J. Non Cryst. Solids.

[56] Aull B, Jenssen H (1982). Vibronic interactions in Nd: YAG resulting in nonreciprocity of absorption and stimulated emission cross sections. IEEE J. Quantum Electron.

[57] Lai S. T. Review of spectroscopic and laser properties of emerald. In *Proc. Volume**0622, High Power and Solid State Lasers*. 146–150 (SPIE, Los Angeles, CA, 1986).

[58] Silfvast W. T. *Fundamentals of Photonics.* pp1–45 (SPIE, Storrs, CT, 2003).

